# LWheatNet: a lightweight convolutional neural network with mixed attention mechanism for wheat seed classification

**DOI:** 10.3389/fpls.2024.1509656

**Published:** 2025-01-10

**Authors:** Xiaojuan Guo, Jianping Wang, Guohong Gao, Zihao Cheng, Zongjie Qiao, Ranran Zhang, Zhanpeng Ma, Xing Wang

**Affiliations:** School of Computer Science and Technology, Henan Institute of Science and Technology, Xinxiang, China

**Keywords:** wheat seed image, wheat seed classification, mixed attention mechanism, lightweight CNN, machine vision

## Abstract

**Introduction:**

With the advent of technologies such as deep learning in agriculture, a novel approach to classifying wheat seed varieties has emerged. However, some existing deep learning models encounter challenges, including long processing times, high computational demands, and low classification accuracy when analyzing wheat seed images, which can hinder their ability to meet real-time requirements.

**Methods:**

To address these challenges, we propose a lightweight wheat seed classification model called LWheatNet. This model integrates a mixed attention module with multiple stacked inverted residual convolutional networks. First, we introduce a mixed attention mechanism that combines channel attention and spatial attention in parallel. This approach enhances the feature representation of wheat seed images. Secondly, we design stacked inverted residual networks to extract features from wheat seed images. Each network consists of three core layers, with each core layer is comprising one downsampling unit and multiple basic units. To minimize model parameters and computational load without sacrificing performance, each unit utilizes depthwise separable convolutions, channel shuffle, and channel split techniques.

**Results:**

To validate the effectiveness of the proposed model, we conducted comparative experiments with five classic network models: AlexNet, VGG16, MobileNet V2, MobileNet V3, and ShuffleNet V2. The results demonstrate that LWheatNet achieves the highest performance, with an accuracy of 98.59% on the test set and a model size of just 1.33 M. This model not only surpasses traditional CNN networks but also offers significant advantages for lightweight networks.

**Discussion:**

The LWheatNet model proposed in this paper maintains high recognition accuracy while occupying minimal storage space. This makes it well-suited for real-time classification and recognition of wheat seed images on low-performance devices in the future.

## 1 Introduction

With the continuous development of global agricultural production, the classification of crop seed varieties has become particularly important in both agricultural scientific research and practical production (Chen et al., 2024). Wheat, one of the most crucial agricultural products worldwide, is cultivated across a vast area globally. It is also a major crop in China’s grain production, with substantial cultivation areas and total output. The stable development of wheat is vital for China’s food security reserves (Jiang et al., 2021). Accurately identifying and classifying different varieties of wheat seeds not only aids in controlling and improving seed quality but also provides a scientific basis for crop management and optimization. In this context, image classification technology, as an efficient and precise tool, is being increasingly utilized in the agricultural sector.

The classification of wheat seed varieties is a crucial aspect of agricultural production, encompassing various stages from seed selection to sowing, fertilizing, and harvesting. Accurate identification of seed varieties is essential at each step. Traditional methods for seed classification primarily rely on manual observation and empirical judgment, which are both time-consuming and labor-intensive, with limited accuracy. However, the rapid advancements in computer vision and machine learning technologies have introduced automated recognition methods based on image classification, offering new possibilities for crop seed variety classification. These advanced methods not only substantially enhance classification efficiency but also significantly improve accuracy, thereby providing robust support for the scientific management of agricultural production.

Traditional machine learning methods, such as Support Vector Machine (SVM), K-Nearest Neighbors (KNN), and Random Forest (RF) (Zhang et al., 2019; Tang et al., 2020; Panda et al., 2023), were widely employed in early research on crop seed variety classification. These methods typically rely on manually extracted features, such as color, shape, and texture, which serve as input data for classification. For instance, the classification of different seed varieties can be achieved by extracting their color and shape features. However, manual feature extraction demands substantial domain knowledge and experience, and it exhibits limitations when dealing with complex and diverse seed grain image data.

In recent years, image classification using deep learning has achieved remarkable results in various fields (Zhang et al., 2024a; Wang et al., 2024; Li et al., 2024; Zhang et al., 2024b). Particularly in the field of agriculture, the application of deep learning techniques has shown great potential and advantages. For example, classical deep learning models such as AlexNet, VGG, and ResNet have been successfully ap-plied to various crop seed variety classification tasks, demonstrating excellent classification performance (Xu et al., 2022; Sabanci, 2023). Traditional crop seed classification methods usually rely on manual feature extraction and rule-based classifiers, which are not only time-consuming and laborious but also have low accuracy and robustness. In contrast, deep learning models can automatically extract and learn the features of different seed types by training on a large number of seed images, thus achieving efficient and accurate classification (Gulzar et al., 2023; Zhang et al., 2023). This not only improves the automation of agricultural production but also provides strong technical support for the quality control and variety improvement of crop seeds.

Although deep learning has made significant strides in crop seed variety classification, several challenges and issues remain. Firstly, deep learning models typically require a large amount of labeled data for training. In the agricultural domain, obtaining large-scale, high-quality labeled data is more challenging, and there are fewer publicly available wheat seed image datasets for deep learning. Secondly, the training and inference processes of deep learning models demand substantial computational resources, which can be difficult to achieve in resource-limited agricultural environments. Our re-search aims to address these challenges, the main contributions of this paper are as follows:

1. We constructed a dataset comprising single-seed images of five different wheat varieties to provide a robust database for subsequent model training and testing.
2. We proposed a lightweight convolutional neural network, LWheatNet, which integrates a mixed attention mechanism with stacked inverse residual convolutional net-work. This model not only enhances the classification performance of wheat images but also maintains a small number of parameters.
3. We designed comparative, classification, and generalization experiments to demonstrate the superiority of our proposed model using various evaluation metrics.

The remainder of this paper is organized as follows. section 2 analyzes the related work; Section 3 introduces the materials and methods; Section 4 illustrates the experimental results and analysis; Section 5 offers the discussion; and finally, Section 6 concludes the paper and provides future prospects.

## 2 Related work

### 2.1 Machine learning-based approach for crop seed classification

The traditional machine learning process for image classification and recognition primarily involves several steps: image preprocessing, feature extraction, feature selection, and classifier construction. Scholars in the agricultural field have achieved notable success using machine learning methods. For instance, Punn et al. (Punn and Bhalla, 2013) and colleagues employed machine learning algorithms to classify and recognize wheat seeds and varieties, achieving an accuracy of 86.8% with Support Vector Machines (SVM) and 94.5% with neural networks. Deng et al. (Deng et al., 2013) and his team used Artificial Neural Networks (ANN) and SVM algorithms to extract 48 features, including morphology, color, and texture, from each sample image. They successfully recognized 83% of wheat varieties in a test dataset comprising 12 varieties. Meng et al. (Meng et al., 2017) and other researchers utilized an improved Back Propagation (BP) network combined with Principal Component Analysis (PCA) for dimensionality reduction, achieving an average recognition rate of 91.58% for wheat varieties. After optimizing with the Particle Swarm Optimization (PSO) algorithm, the recognition rate increased to 94.3%. Zhu et al. (Zhu et al., 2019) and colleagues combined spectroscopy and im-age-based detection techniques to identify crop seed varieties, employing five different classifiers and achieving an identification accuracy of 99.8%. Ali et al. (Ali et al., 2020) employed various classification models, including Random Forest (RF), BayesNet (BN), LogitBoost (LB), and Multilayer Perceptron (MLP), to classify corn seeds. In their comparative analysis of these four machine learning classifiers, the MLP demonstrated outstanding classification accuracy, achieving 98.93% on regions of interest (ROIs) sized 150 x 150 pixels. Specifically, the MLP achieved accuracy values of 99.8% for Desi Makkai, 97% for Sygenta ST-6142, 98.5% for Kashmiri Makkai, 98.6% for Pioneer P-1429, 99.9% for Neelam Makkai, and 99.4% for ICI-339. Feng et al. (Feng et al., 2022) extracted color, morphological, and texture features from wheat grains, resulting in a total of 28 feature values. they constructed various feature fusion models, as well as data degradation and data enhancement models. Experimental results demonstrated that the average recognition accuracy based on the fusion of the three feature sets—texture, morphology, and color—was 91.02%. Nansen et al. (Nansen et al., 2022) used Linear Discriminant Analysis (LDA) and SVM to classify crop seeds. Their experimental results demonstrated that the classification accuracy of both LDA and SVM de-creased linearly in response to the introduction of object assignment error and the experimental reduction of spectral repeatability. Bhavana et al. (Bhavana and Jayaraju, 2024) utilized the SVM and Random Forest (RF) algorithms to identify boundaries within classified crop areas. The pro-posed algorithm yielded impressive results with a high level of accuracy.

In conclusion, traditional machine learning methods require manual selection and extraction of features from images, which lack adaptivity and make it difficult to model complex data structures. This limitation affects the performance of machine learning algorithms. In practical applications, determining which features are useful is challenging. Currently, the identification of different wheat varieties primarily relies on manual methods, where people observe the morphology, color, size, and other features of wheat grains to differentiate between varieties. This manual identification process is labor-intensive and requires extensive practical experience, thereby limiting its application in production practices.

### 2.2 Deep learning-based approach for crop seed classification

Deep learning-based image classification and recognition methods primarily include Convolutional Neural Networks (CNN), Recurrent Neural Networks (RNN), with CNN being one of the most widely used techniques. In recent years, deep learning recognition methods have become significant in the field of image recognition and have been extensively applied to the study of crop species (Qiu et al., 2019). Chen et al. (Chen et al., 2018) utilized a deep convolutional network for the automatic extraction of wheat features, achieving an average accuracy of 97.78% in wheat variety recognition tasks. The classification confusion matrix exhibited a diagonal array trend, indicating high accuracy. Xie et al. (Xie et al., 2020) proposed an algorithm for recognizing the integrity of oil tea seeds using convolutional neural networks. By employing techniques such as network simplification and hyper-parameter optimization, the optimized network achieved an accuracy of 98.05% in recognizing the integrity of oil tea seed grains. Chen et al. (2021) introduced an attention mechanism into the model, which enhanced the model’s focus on important features, thereby improving recognition performance. This method was successfully applied to rice pest image recognition tasks, achieving a recognition rate of up to 99.67% on a public dataset, with experiments vali-dating the procedure’s effectiveness. Javanmardi et al. (Javanmardi et al., 2021) proposed a novel approach for recognizing and classifying maize seeds using a deep convolutional network as a feature ex-tractor. When compared to traditional classifiers, the deep convolutional neural network approach achieved a recognition accuracy of 98.2% for maize seeds. Wu et al. (Wu et al., 2021) de-signed three deep neural networks with typical structures based on a sample-rich pea dataset, achieving the highest accuracy of 99.57%. The VGG model was then transferred to classify four target datasets (rice, oat, wheat, and cotton) with limited samples. The accuracies of the deep transferred model on these four datasets were 95%, 99%, 80.8%, and 83.86%, respectively. Luo et al. (Luo et al., 2023) and colleagues utilized deep learning to efficiently classify weed seeds by selecting appropriate classification models. This approach is crucial for effective weed management and control. Tugrul et al. (Tugrul et al., 2023) developed an improved Convolutional Neural Networks (CNNs) model by adding new layers to the final layers of well-known CNN architectures from the literature. The accuracy of this custom CNN model for seed classification ranged between 91% and 94%. Li et al. (Li et al., 2023) used VGG16, ResNet-50, Inception-V3 and other convolutional neural networks to establish a wheat seed variety identification and classification model through migration learning, and recognized six types of wheat varieties, and the highest recognition accuracy of the validation set was 99.35%. Que et al. (Que et al., 2023) proposed a convolutional neural network (CR-CNN) based on envelope removal based on the problem of detection of large-volume seeds, and showed that the classification accuracy could reach 96.125% after using the envelope removal method. For the problem of hyperspectral image detection efficiency is difficult to meet the large number of seed detection, based on the envelope removal - convolutional neural network (CR-CNN) is proposed, and the study shows that after using the envelope removal method, the classification accuracy can reach up to 96.125%. Wang et al. (Wang et al., 2023) proposed a novel deep learning approach for multi-scenario crop classification called Cropformer. The results demonstrated that Cropformer could build up *a priori* knowledge using unlabeled data and learn generalized features using labeled data, making it applicable to crop classification in multiple scenarios. Zhang et al. (2024c) proposed a wheat variety identification framework called generate adversarial-driven cross-aware network (GACNet), comprising a semi-supervised generative adversarial network for data augmentation and a cross-aware attention network for variety identification, experiments result demonstrate that the GACNet outperforms state-of-the-art methods for wheat variety identification. Gill et al. (Gill et al., 2024) used deep learning techniques such as CNN, RNN, and LSTM to classify wheat crops, with test accuracy results ranging around 85%. Sable et al. (Sable et al., 2024) introduced a lightweight soybean seed defect identification network (SSDINet). Experimental results demonstrated that SSDINet achieved the highest accuracy of 98.64%, with 1.15 million parameters in 4.70 milliseconds, surpassing existing state-of-the-art models.

In summary, it is evident from the findings of numerous scholars that deep learning-based methods, particularly convolutional neural networks have significantly advanced crop seed classification, achieving high accuracy and efficiency. Despite the considerable progress made by many researchers, several challenges remain:

1. The agricultural domain lacks comprehensive image datasets of various wheat varieties, hindering downstream research;
2. The performance of crop seed image classification still requires improvement;
3. The computational resource demands of current deep learning models remain high. Therefore, the development of lightweight image classification models is imperative to meet the needs of agricultural experts and farmers. Such lightweight models can operate on resource-constrained devices and significantly reduce computational costs and time consumption while maintaining classification accuracy. This advancement will facilitate the broader application of image classification technology in actual agricultural production, enhance work efficiency, and promote the development of intelligent agriculture.

## 3 Materials and methods

### 3.1 Data construction

#### 3.1.1 Image acquisition

Collected wheat varieties were photographed as single seeds from multiple angles to build a comprehensive wheat seed image dataset. The images were captured from two angles: groin up and groin down, to improve the compatibility and accuracy of variety identification. A SangNond2K measurement electron microscope paired with an industrial camera was used for seed imaging. For each variety, 500 fully developed seeds were selected and prepared for single-seed image collection. The collection equipment parameters were set as **Table 1**.

**Table 1:** Equipment parameters.

Equipment	Parameters
Equipment model	SN0745-60U2K
CCD camera	1600 pixel 2K industrial camera
Auxiliary eyepiece	0.5X
Zoom objective	3.5
Main objective magnification	5.0
Electronic magnification	180
Working distance	15 cm
Light source	LED ring spotlight

**Figure 1:**
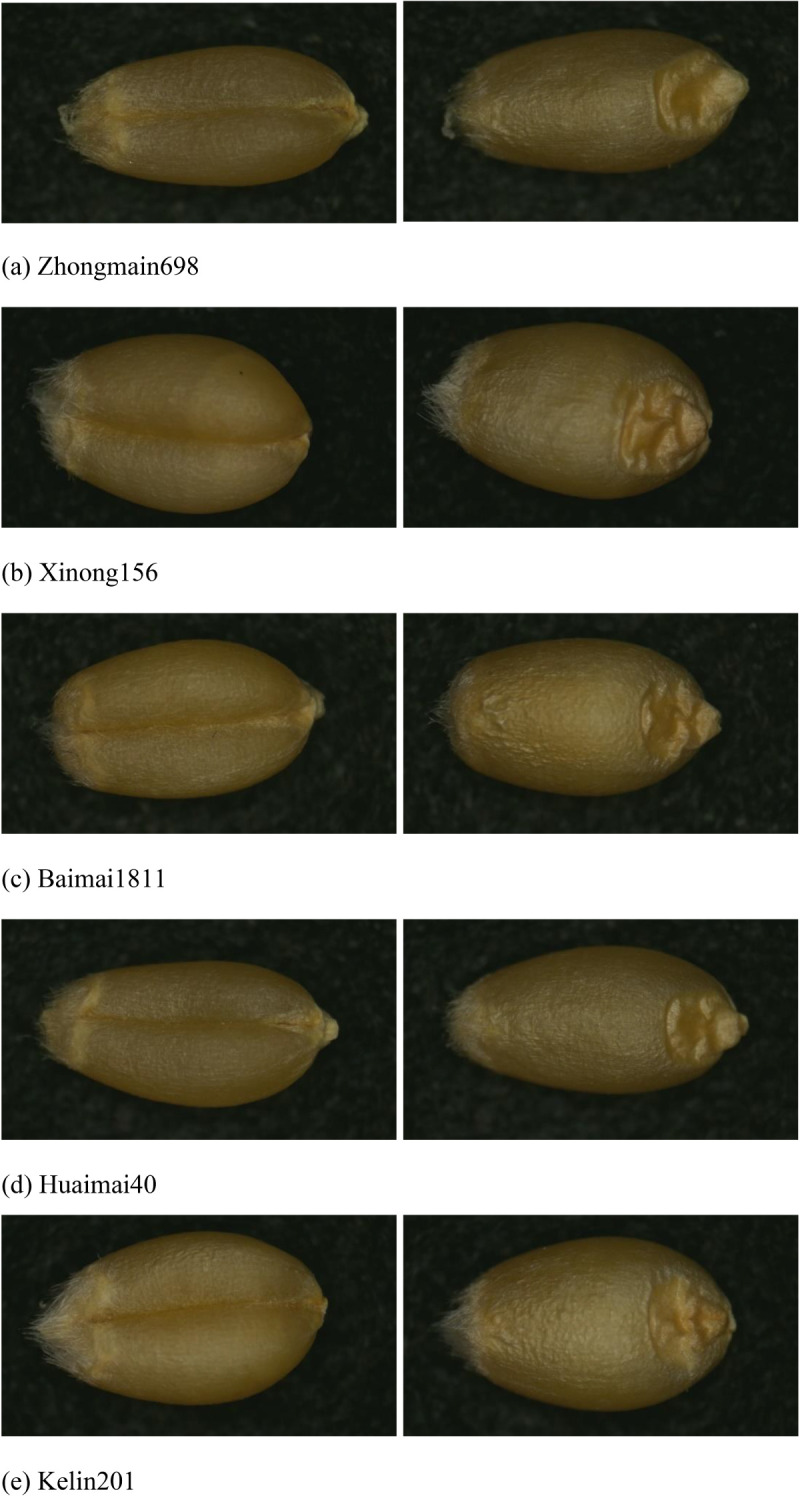
Original images of 5 types of wheat varieties. **(A)** Zhongmain698, **(B)** Xinong156, **(C)** Baimai1811, **(D)** Huaimai40, **(E)** Kelin201.

Photographs were taken against a black light-absorbing flannel background, with a resolution of 1920 × 1080 pixels. Automatic white balance (AWB) and wide dynamic range (WDR) were turned off, and the LED fill light was set to medium. Images were captured class by class according to the seed variety, and each category was saved in a folder named after the variety. This process resulted in 5004 photographs across five varieties. **Figure 1** shows the original images of the collected wheat kernels.

#### 3.1.2 Construction the wheat dataset

**Figure 2:**
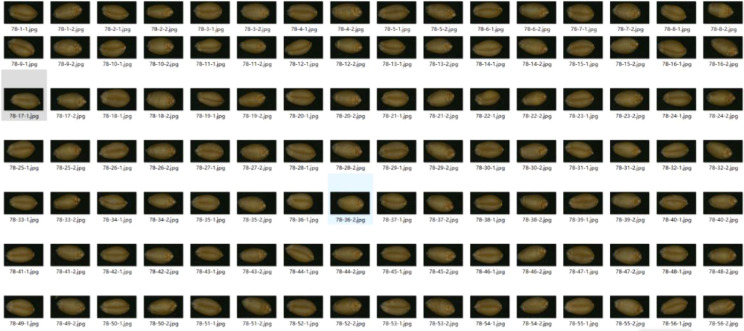
Partial image of Xinong156.

The original images captured in this study were initially named using a format that combined the time of capture with an automatic numbering system, which did not provide information regarding the variety category or the shooting angle of the wheat. To address this limitation, we renamed the original image files and organized them into folders designated by their respective varieties. The new naming format for the image files is structured as follows: variety number_particle number_shooting angle (where 1 indicates groin up and 2 indicates groin down). For instance, for the Xinong 156 variety, if the 78th grain was photographed groin up as the third grain, the image would be named 78-3-1. In total, 1,000 images representing both shooting angles for each variety were saved in the Xinong 156 folder. This naming convention was consistently applied across all five varieties. **Figure 2** illustrates several images of the Xinong 156 variety.

**Figure 3:**
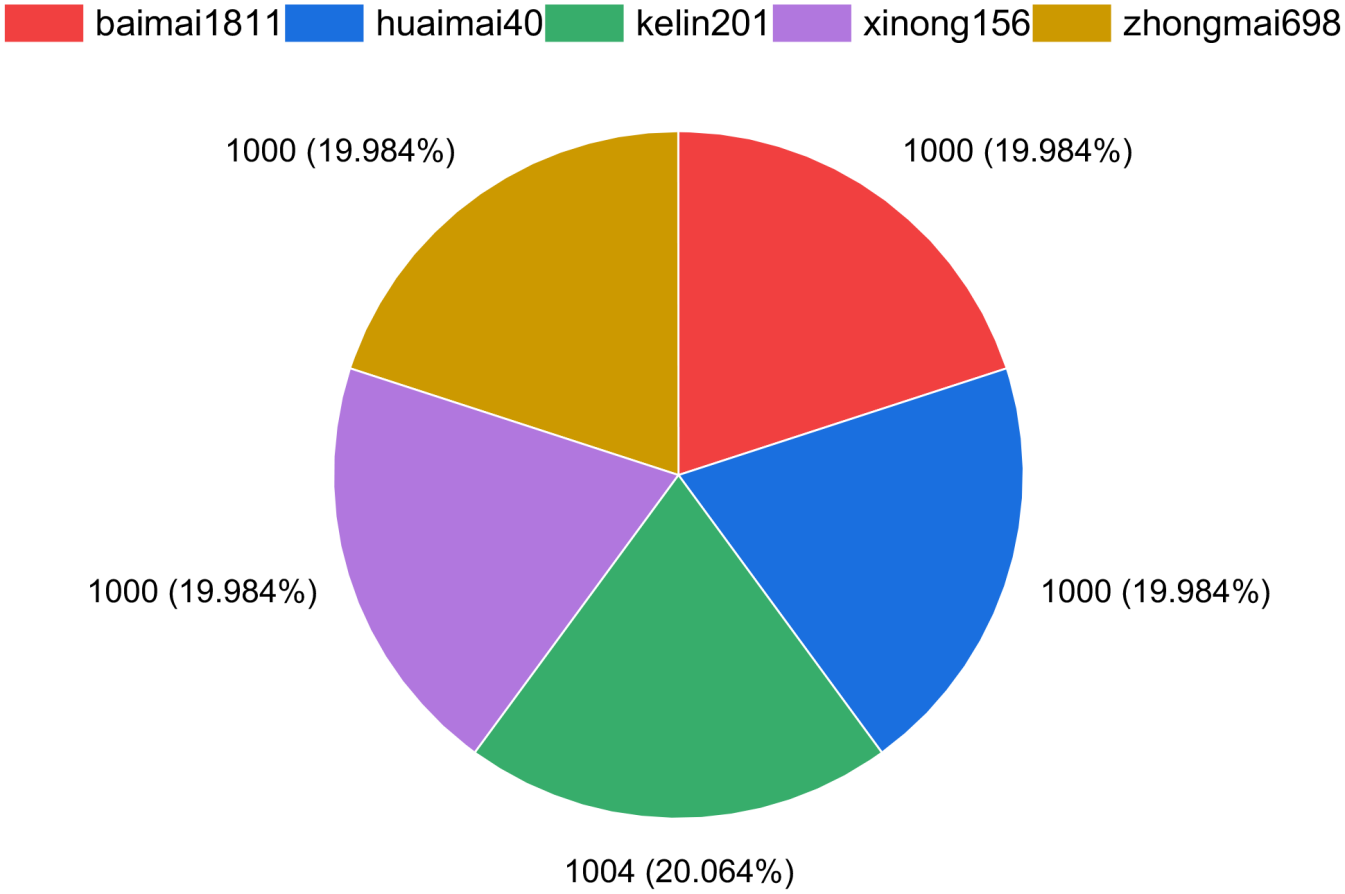
Distribution of the wheat dataset.

A dataset of wheat grains has been constructed based on five categories of wheat that have undergone preprocessing, as illustrated in **Figure 3**. From **Figure 3**, it can be observed that the images of single wheat grains across the five categories are evenly distributed. This uniformity is beneficial for enhancing the generalization capability of the models, helping to mitigate potential biases that could arise from imbalanced data. The balanced distribution of the dataset provides solid support for the subsequent training and validation of related models, allowing them to effectively learn the characteristics of each wheat type.

In addition, the dataset has been carefully developed with an emphasis on diversity and representativeness, ensuring that samples from each wheat category exhibit a range of variability in morphology, color, and texture. This inherent diversity is expected to enhance the robustness of the models, thereby improving their accuracy and reliability in practical applications. The experiments detailed in Section 4 will utilize this dataset for the training, tuning, and evaluation of the models, with the goal of facilitating efficient recognition and classification of different types of wheat grains. Through this approach, we aim to contribute valuable scientific insights and technical support for wheat quality assessment and cultivation management.

The constructed wheat dataset was thoughtfully partitioned into training, validation, and test sets, adhering to a ratio of 8:1:1. The results of this division are illustrated in **Table 2**. This organized approach is designed to promote a balanced representation of the data across the various sets, ultimately supporting robust model development and evaluation.

**Table 2:** Partitioning of the wheat dataset.

Category	Training	Validation	Test	Total
baimai1811	801	100	99	1000
huaimai40	801	100	99	1000
kelin201	804	100	100	1004
xinong156	801	100	99	1000
zhongmai698	801	100	99	1000
total	4008	500	496	5004

To accommodate the diverse input requirements of various models, the images in the training set were subjected to random cropping and normalization. In contrast, the validation and test sets underwent normalization only. This approach ensures that the training data is well-prepared while maintaining consistency in the evaluation process for the validation and test sets.

### 3.2 Model of LWheatNet

#### 3.2.1 The architecture of LWheatNet

**Figure 4:**
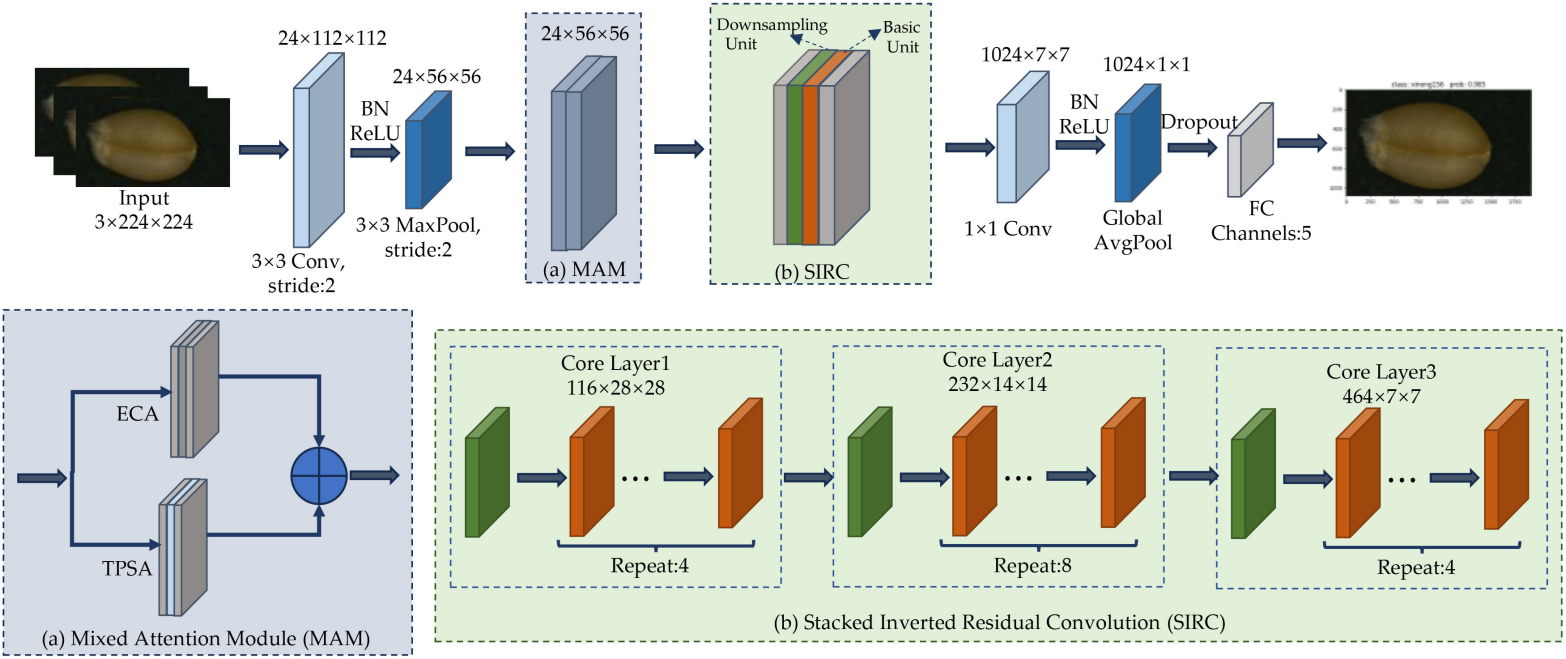
Structure of LWheatNet.

To further improve classification accuracy while maintaining a lightweight model, this paper proposes LWheatNet, a lightweight classification model. The LWheatNet model is depicted in **Figure 4**. The core part of LWheatNet include a Mixed Attention Module (MAM) and Stacked Inverted Residual Convolution (SIRC) layer designed to extract wheat seed features. The SIRC significantly reduces computational overhead and the number of parameters by incorporating channel split operations, depthwise separable convolutions, and channel shuffling. Considering the characteristics of the wheat image dataset—such as a uniform background and subtle feature differences—the model also integrates a MAM. LWheatNet fully accounts for both local and global features of wheat seed images, thereby enhancing classification accuracy under limited computational resources.

As shown in **Figure 4**, the LWheatNet architecture consists of the following components: an input layer, a 3x3 convolutional layer, a max pooling layer, a MAM layer, a SIRC layer, a 1x1 convolutional layer, a global average pooling layer, a fully connected layer, and an output layer.

The MAM parallelizes channel attention and spatial attention, while the SIRC consists of three stacked core layers, each containing a downsampling unit and multiple basic units. The specific execution process of the LWheatNet is as follows. First, the input, a 224×224×3 image, undergoes a 3×3 convolution operation, producing an output feature map of size 112×112×24. This feature map is then reduced to 56×56×24 by a 3×3 max pooling layer. Subsequently, the basic and downsampling units in the SIRC are repeatedly stacked through three layers: Core Layer 1, Core Layer 2, and Core Layer 3. Following a 1×1 convolution and global average pooling, the number of feature map channels is reduced to 1,024. Finally, the output is set to 5 through a fully connected (FC) layer.

#### 3.2.2 Layers of LWheatNet

1. Input Layer

The LWheatNet model processes wheat seed image as input. Receive the input image and automatically crop it to 224×224. Perform normalization to standardize the input image, making it suitable for subsequent processing.

2. 3x3 convolutional Layer

A 3x3 convolutional layer with 3 convolutional kernels, 24 output channels, and a stride of 2 is applied. This convolution is followed by normalization and a ReLU activation function for the initial extraction of low-level features, such as edges and textures, to reduce the spatial resolution of the wheat seed image.

3. Max Pooling Layer

A 3x3 max pooling layer with a stride of 2 reduces the size of the feature map and lowers the computational complexity.

4. Mixed Attention Module Layer

This layer integrates channel attention and spatial attention in parallel. The channel attention employs Efficient Channel Attention (ECA), which more effectively focuses on the representative features of wheat grain images. Conversely, spatial attention utilizes Two-Path Spatial Attention (TPSA), which uses convolutional kernels of two different scales to extract detailed features from the wheat grain images. In this study, convolutional kernels of sizes 3x3 and 5x5 are used to extract features, which are subsequently fused by summing.

5. The Stacked Inverted Residual Convolution Layer

The Stacked Inverted Residual Convolution Layer (SIRC) comprises three core layers, each containing multiple basic units and a downsampling unit. Each core layer employs operations such as channel splitting, depthwise separable convolution, channel shuffling, and residual linking to capture more detailed features of wheat grain images. The use of grouped convolution and depthwise separable convolution significantly reduces the computational load, while channel shuffling enhances the interaction between different features, thereby improving feature representation capabilities. Residual linking is utilized to preserve the information in the input feature map and prevent gradient vanishing.

6. 1x1 Convolution Layer

The 1x1 convolution layer performs nonlinear transformation through 1x1 convolutions, providing further feature extraction. It is primarily used to adjust the number of channels in the feature map, preparing it for subsequent global pooling and fully connected layers.

7. Global Pooling Layer

This layer performs global average pooling on the feature map, reducing it to a global feature vector and decreasing the number of parameters.

8. Fully Connected Layer

The FC performs a linear transformation on the input feature vectors, mapping the high-dimensional features to the desired dimensions of the classifier. This transformation renders the feature vectors suitable for classification purposes.

9. Output Layer

The output from the Fully Connected Layer is forwarded to the Softmax layer, whose primary role is to transform these outputs into probability distributions for each class. The Softmax function achieves this by applying an exponential operation to each element of the input vector and then normalizing the results so that the sum of all output probabilities equals 1. This resulting probability distribution indicates the likelihood that the input text belongs to each class. The class with the highest probability is deemed the final classification result of the model. By performing exponential operations on the input values and normalizing them, the Softmax function ensures that the output values represent valid probability distributions.

#### 3.2.3 Loss function and optimizer

During model training, the cross-entropy loss function is used to measure the difference between the predicted categories and the actual categories. This function calculates the negative log-likelihood of the model’s predictions, guiding the optimization of the model parameters. By assessing the divergence between predicted probabilities and true labels, the cross-entropy loss function provides a measure of model performance. LWheatNet employs this loss function to quantify the discrepancy between its outputs and the true labels, as defined in (1):


(1) $$CroessEntropy\left(y,\hat{y}\right)=-\sum_{i=1}^{N}y_{i}\times log\left({\hat{y}}_{i}\right)$$

Where *N* is the number of categories in the dataset, and $y_{i}$ means the code of the true label category. ${\hat{y}}_{i}$ is the distribution probability of the label category. $R_{Loss}$ is the average value of the loss function for each sample, as shown in (2).


(2) $$R_{Loss}=\frac{1}{N}\sum_{j=1}^{N}R_{j}\left(y,\hat{y}\right)$$

where *N* is the number of samples in the dataset.

The optimization of model parameters is carried out using the Momentum stochastic gradient descent (SGD) optimizer. The momentum SGD detailed as (3) to (5):


(3) $$g=\nabla_{\theta_{k-1}}L\left(\theta \right)$$

(4) $$V_{t}=\beta V_{t-1}+\left(1-\beta \right)g$$

(5) $$\theta_{k}=\theta_{k-1}-\eta w_{k}$$

where $\theta_{k-1}$ refers the model parameter vector at step *k-1*, $\nabla_{\theta_{k-1}}L\left(\theta \right)$ represents the gradient of the loss function *L* with respect to the parameter $\theta_{k-1}$ , *η* is the learning rate, and *β* shows the momentum parameter, which is set to 0.9 in this paper. $w_{k}$ denotes the momentum, indicating the weighted accumulation of historical gradients.

In the process of using the gradient descent algorithm to optimize the objective function, when the loss value of the objective function is approaching the global optimal solution, the update step size should be reduced. This allows the objective function to get closer to the global optimal solution. This technique is known as the learning rate decay strategy. Various methods exist for adjusting the learning rate, including equal interval adjustment, exponential decay adjustment, cosine annealing adjustment, adaptive adjustment, and customized adjustment. In this paper, we use cosine annealing adjustment as the learning rate decay strategy.

The cosine annealing algorithm reduces the learning rate using the cosine function. Initially, the cosine function decreases slowly to determine the correct direction of optimization, and then it decreases rapidly to accelerate the convergence of the function. If the gradient descent algorithm falls into a local optimal solution during the training process, the second half of the cosine function can be used to suddenly increase the learning rate. This helps to jump out of the local optimal solution and find the path to the global optimal solution. This method is a stochastic gradient descent approach with restarts. The cosine annealing algorithm is represented in Equation 6.


(6) $${\text{η}}_{t}={\text{η}}_{min}^{i}+\frac{\left({\text{η}}_{max}^{i}-{\text{η}}_{min}^{i}\right)\left(1+cos\left(\frac{T_{cur}}{T_{i}}\pi \right)\right)}{2}$$

where the *i* denotes the index, ${\text{η}}_{max}^{i}$ , ${\text{η}}_{min}^{i}\;$ represent the range of the learning rate, which we set to 1 and 0.01, respectively., $T_{cur}$ indicates the current epoch, while $T_{i}$ refers to the epochs of *i-th* cycle.

By leveraging the combined influence of the cross-entropy loss function and the SGD optimizer, the model persistently adjusts and fine-tunes its parameters to boost classification accuracy and stability. These adjustments take place during the training phase, ensuring a gradual enhancement in the model’s classification performance.

### 3.3 Mixed attention module

**Figure 5:**
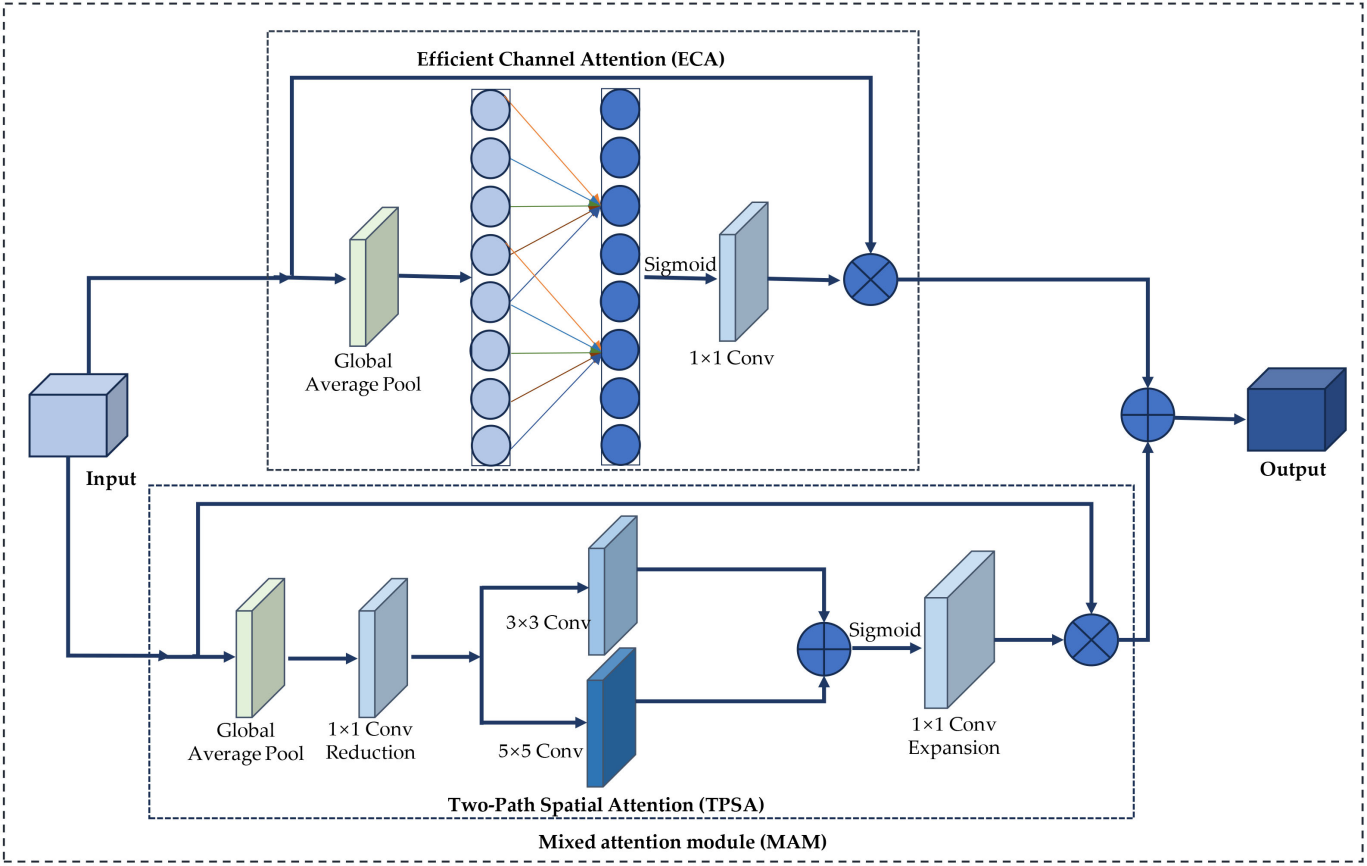
Structure of MAM.

The attention mechanism in deep learning processes data by selectively focusing on relevant elements, automatically learning and calculating each input’s contribution to the output. In the task of wheat seed classification, the network model extracts feature information through a series of convolution operations. However, the impact of different feature information on distinguishing various types of wheat seeds is not uniform. The attention mechanism can process information from different regions of the input image differently. To enhance the network model’s focus on specific regions and reduce interference from other factors in wheat seed recognition, we build upon the Convolutional Block Attention Module (CBAM) (Woo et al., 2018), which uses a serial connection between channel and spatial attention modules. This serial connection may affect the feature ex-traction capability of subsequent modules to a certain extent. To address this issue, this paper proposes a Mixed Attention Module (MAM), which connects the channel attention and spatial attention modules in parallel, ensuring that the two modules do not interfere with each other, and ultimately performs feature fusion. The structure of MAM is shown in **Figure 5**.

As depicted in **Figure 5**, the Mixed Attention Module (MAM) comprises channel attention and dual-path spatial attention modules. The following sections will provide a detailed explanation of these two attention modules.

1. Channel attention module

The classification and identification of wheat grain images are challenging due to the relatively uniform backgrounds and subtle differences among various wheat varieties. However, the channel attention module effectively emphasizes the most representative features within the wheat grain images, thereby enhancing the classification process. Wang et al. (Wang et al., 2020) proposed an Efficient Channel Attention (ECA) mechanism, which facilitates local cross-channel interactions without dimensionality reduction. This approach utilizes one-dimensional convolution for cross-channel interactions, resulting in reduced algorithmic complexity while simultaneously enhancing network performance with only a minimal increase in the number of parameters. In this study, the channel attention within the mixed attention module is based on the ECA mechanism. The operational workflow of ECA is detailed as follows.

Given an input $X\in R^{H\times W\times C}$ which is transformed to $X\in R^{1\times 1\times C}$ through global average pooling, the resulting feature map *y* is then reshaped to $X\in R^{C\times 1}$ to meet the requirements of the subsequent convolution operation. This is accomplished through the application of one-dimensional convolution, utilizing the associated one-dimensional convolution weights. The calculations are defined in Equations 7-10.


(7) $$y=\frac{1}{H\times W}\sum_{i=1}^{H}\sum_{j=1}^{W}F_{i,j}\left(x\right)$$

(8) $$w_{i}=\sigma \left(\sum_{j=1}^{k}w_{i}^{j}y_{i}^{j}\right),y_{i}^{j}\;\in \;\Omega_{i}^{k}$$

(9) $$w=\sigma \left(C1D_{k}\left(y\right)\right)$$

(10) $$k=\varphi \left(C\right)={\left|\frac{log_{2}\left(C\right)}{\gamma }+\frac{b}{\gamma }\right|}_{Odd}$$

Where, H, W and C represent the height, width, and number of channels of the input image, respectively. $\Omega_{i}^{k}$ denotes the set of k neighboring channels of $y_{i}$ . C1D stands for one-dimensional convolution, *k* is the convolution kernel, *σ* is the sigmoid function, ${\left|t\right|}_{Odd}$ denotes the nearest odd number to *t*, and *b* and *γ* are constants with values of 1 and 2, respectively.

The output is subsequently processed through a Sigmoid activation function. Following this, the normalized output undergoes a dimensional transformation to yield $X\in R^{1\times 1\times C}$ . Finally, the channel attention weights derived from the previous step are multiplied with the original feature map of the input, resulting in the final output.

2. Two-Path Spatial Attention

As shown in **Figure 5**, TPSA is constructed through operations such as global average pooling, dimensionality reduction, convolutions of different scales, feature fusion, dimensionality expansion, activation, and element-wise multiplication.

First, each channel of the input feature map *F* undergoes global average pooling to obtain a global feature vector *G*. The global average pooling layer effectively captures global information and reduces the spatial dimensions of the feature map. Then, a 1×1 convolution is used to reduce the dimensionality of the global feature vector *G*, thereby reducing the number of channels. In this paper, the number of channels is reduced to 1/16 of the original.

Subsequently, the feature map *G*′ is subjected to convolution operations using multiple kernels of varying sizes, specifically 3×3 and 5×5 convolution kernels. These two convolution paths are designed to capture features at different scales, thereby enhancing the diversity of feature extraction and improving the model’s expressive capability. The feature maps *P*1and *P*2 obtained from the two convolution paths, are then combined through a straightforward summation operation.

Third, a 1×1 convolution is used to expand the dimensions of the fused feature map, and the expanded feature map *P*′ undergoes Sigmoid activation to obtain the attention weights *A*. The Sigmoid function primarily normalizes the values of the feature map. The purpose of dimensionality expansion is to restore the original number of channels to maintain consistency with the input feature map.

Finally, the original input feature map *F* is element-wise multiplied by the attention weights *A* to produce the final output feature map *F*′.

The MAM is designed to significantly enhance the feature extraction capabilities of wheat seed images by integrating the ECA and TPSA mechanisms in parallel. This innovative approach involves summing the outputs of both ECA and TPSA, thereby capitalizing on the unique strengths of each attention mechanism. The detailed implementation process of the MAM is presented in **Algorithm 1**, which outlines the sequential steps involved in the integration and application of these attention techniques.

Algorithm 1 **The mixed attention module algorithm.**

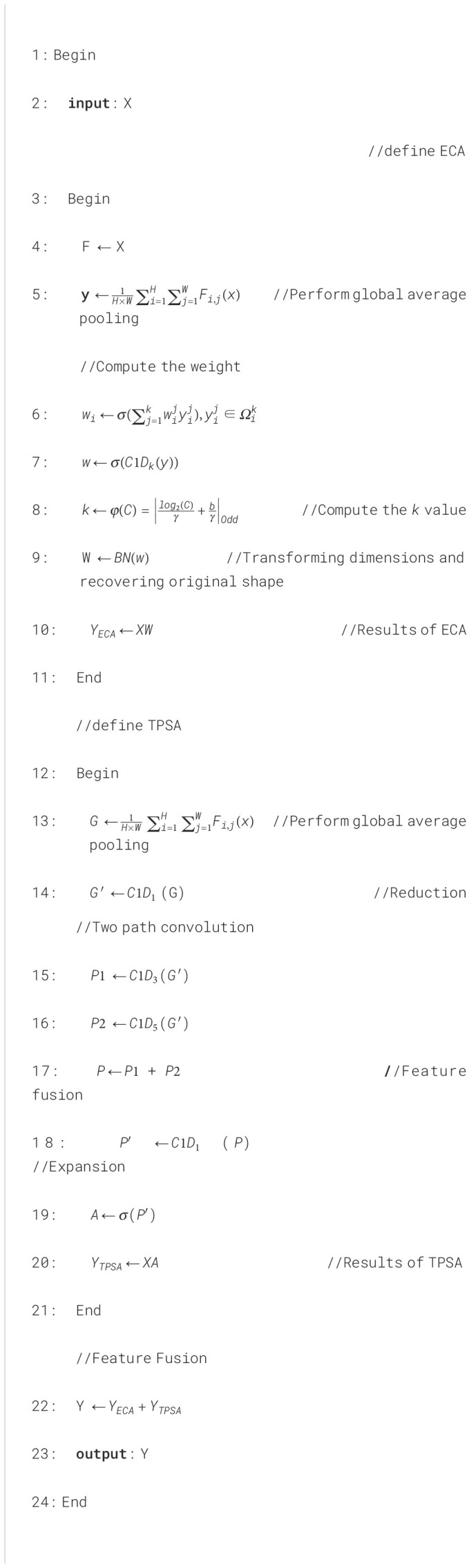

### 3.4 The stacked inverted residual convolution

**Figure 6:**
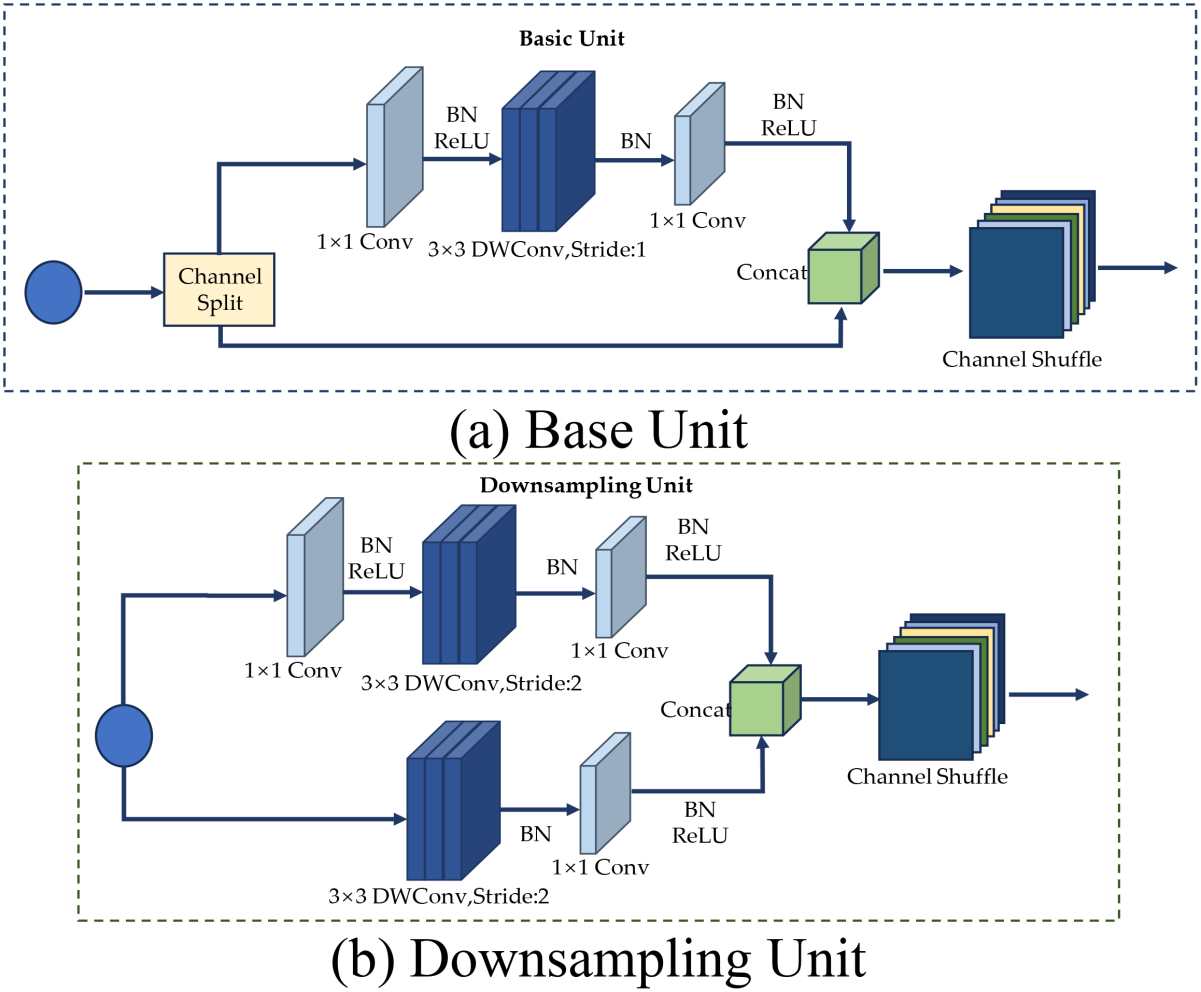
The structure base and downsampling unit. **(A)** Base Unit, **(B)** Downsampling Unit.

The SIRC layer of LWheatNet utilizes three stacked core layers, each comprising two different unit structures, as shown in **Figure 6**. One of the structures is a base unit with a stride of 1, while the other is a downsampling unit with a stride of 2. The main branch of both structures consists of three convolutional layers: 1 × 1 ordinary convolution, 3 × 3 depthwise separable convolution, and 1 × 1 ordinary convolution. However, the composition of the inputs and the side branches differ between the two structures.

In the case of a stride of 1, the base unit structure is used, as shown in **Figure 6A**. This structure first divides the input channels of the feature map equally into two halves using the Channel Split operation. One branch remains unchanged, while the other branch undergoes a series of operations: it first passes through a 1 × 1 ordinary convolution, followed by a Batch Normalization (BN) layer and a ReLU activation function. Next, it goes through a 3 × 3 depthwise separable convolution and another BN layer. Finally, it completes the sequence with another 1 × 1 ordinary convolution, a BN layer, and a ReLU activation function.

The feature matrices from the two branches are then concatenated, and the final output is obtained by channel rearranging the resulting feature matrices. The convolution operations on the right branch of this base unit are simplified compared to those in the ShuffleNet V1 base unit, reducing structural fragmentation. Additionally, pointwise convolution is used instead of pointwise group convolution to lower memory access costs.

Furthermore, the three convolution operations maintain the same number of input and output channels, which enhances the network’s speed. After the convolution operations, the connection and channel rearrangement performed by the two branches can be merged with the Channel Split of the next cell, forming an element-level operation. This reduction in element-level operations improves computational efficiency.

In the case of a stride of 2, the downsampling unit structure is used, as shown in **Figure 6B**. The left branch first passes through a 3 × 3 depthwise separable convolution and a Batch Normalization (BN) layer with a stride of 2. It then proceeds through a 1 × 1 ordinary convolution, another BN layer, and a ReLU activation function. The right branch structure is similar to the basic unit, with the key difference being that the 3 × 3 convolution is performed with a stride of 2. Finally, the two branches are concatenated, and a channel rearrangement operation is performed to obtain the final output.

The different branches in the two units mentioned above use depthwise separable convolution and channel shuffle. Depthwise separable convolution decomposes the standard convolution into two smaller operations: depthwise convolution and pointwise convolution. Depthwise convolution applies the convolution kernel independently to each input channel, while pointwise convolution uses a 1×1 convolution to combine the outputs of these channels. This method significantly reduces the amount of computation and the number of parameters. The channel shuffle operation rearranges the channels of the feature map within the network to facilitate information exchange between the channels. This operation is usually performed after channel splitting to ensure that features from different branches can be effectively fused together, thereby improving the expressive power of the network.

The execution of the basic and downsampling units is shown in **Algorithm 2**.

Algorithm 2 **The execution of the basic and downsampling units.**

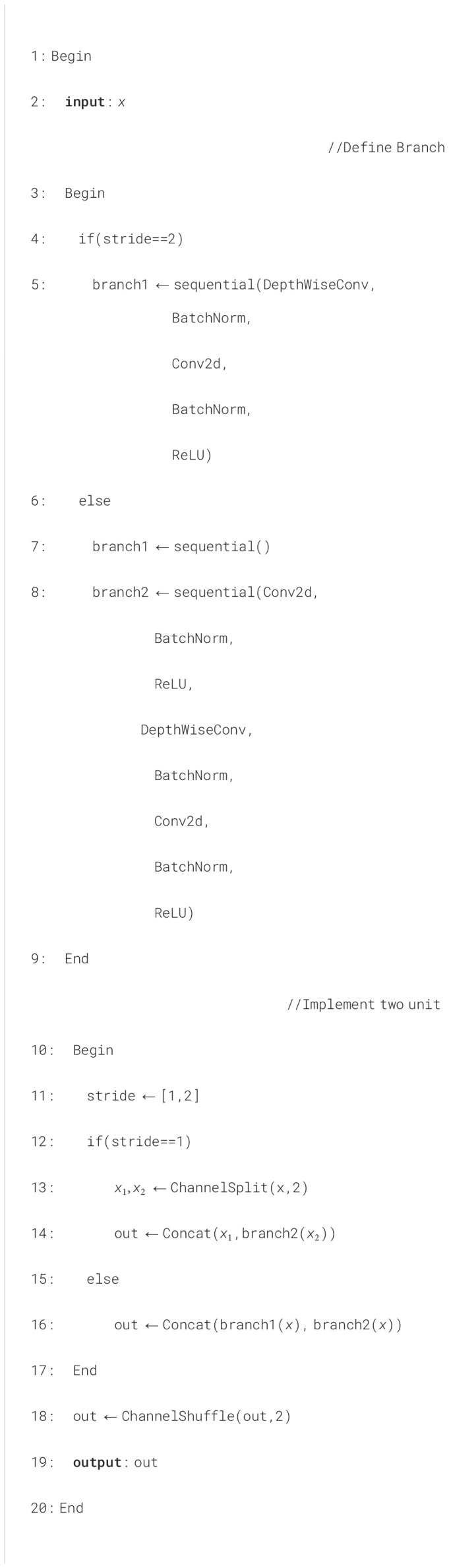

## 4 Results

We utilize Python 3.8 and CUDA 11.7 within the PyCharm 2021 environment, running on a 64-bit Windows 10 operating system. Our hardware setup includes an Intel Xeon^®^ W-2255 CPU and an NVIDIA RTX A4000 GPU with 16GB of GDDR6 memory. We made extensive use of the PyTorch framework and the Torchvision library, as well as various other image processing tools. This capability enabled us to construct, modify, and debug our model in real-time, thereby facilitating the development of an efficient wheat image classification system.

### 4.1 Parameter settings

In this study, we employed a lightweight wheat seed image classification model, LWheatNet. The main parameter settings for the LWheatNet are shown in **Table 3**.

**Table 3:** LWheatNet parameter settings.

Parameters	Value	Parameters	Value
Data_Size	224×224	Loss Function	Cross-Entropy
Batch_Size	16	Conv Kernel	1×1, 3×3
Momentum	0.9	Optimizer	SGD
Weight_Decay	4e-05	Learning_Rate	3e-03
Epoch	50	Dropout	0.4
Activation Fuction	ReLU	Feature Dimension	1024

### 4.2 Evaluation metrics

Precision, recall, F1 score, and accuracy are employed as evaluation metrics for our model. The calculation formulas are provided in Equations 11-14.


(11) $$Precision=\frac{TP}{TP+FP}$$

(12) $$Recall=\frac{TP}{TP+FN}$$

(13) $$F1=\frac{2\times precision\times recall}{precision+recall}$$

(14) $$Accuracy=\frac{TP+TN}{TP+TN+FN+FP}$$

Where, “true positive” (TP) refers to the count of samples where the actual positive instances are correctly identified as positive. “False negative” (FN) signifies the instances where positive cases are mistakenly classified as negative. “False positive” (FP) is used for instances where negative cases are wrongly labeled as positive. “True negative” (TN) denotes the instances where negative cases are accurately classified as negative.

When tackling multi-category classification tasks, it is crucial to thoroughly evaluate the performance of the classification model using metrics such as macro average, micro average, and weighted average. Macro averaging evaluates the model’s performance across all categories by calculating the arithmetic mean of precision, recall, and F1 score for each category. Micro averaging combines the prediction results of all categories to derive overall evaluation metrics. The weighted average addresses sample size imbalances across categories by multiplying each category’s metrics by the proportion of its sample size relative to the total, and then calculating a weighted mean. In this study, weighted averages are employed to evaluate the performance metrics of multiple comparative models on the wheat seed dataset. The formulas for weighted average precision (Precision_w_), weighted average recall (Recall_w_), and weighted average F1 score (F1_w_) are provided in Equations 15-18.


(15) $$Precision_{w}=\sum_{i}w_{i}P_{i}$$

(16) $$Recall_{w}=\sum_{i}w_{i}R_{i}$$

(17) $$F1_{w}=\frac{2\times Precision_{w}\times Recall_{w}}{Precision_{w}+Recall_{w}}$$

(18) $$w_{i}=\frac{S_{i}}{S}$$

where *S_i_* represents the number of *i*-th category, and S denotes the total number of samples; *P_i_* and *R_i_* are the precision and recall for the *i*-th category, respectively.

### 4.3 Ablation experiments

LWheatNet is built upon the foundational WheatNet network module and incorporates attention components such as Two-Path Spatial Attention (TPSA) and Efficient Channel Attention (ECA). To assess the significance of each component within LWheatNet, we conducted a series of ablation studies:

1. WheatNet: The core layer of WheatNet consists of stacked inverted residual convolutions, which include multiple downsampling units and several basic units. This architecture is designed to classify wheat images across training, validation, and test datasets.
2. WheatNet+TPSA: Building upon WheatNet, the TPSA (Two-Path Spatial Attention) module is incorporated to enhance the model’s ability to focus on important spatial features. (3)WheatNet+TPSA+ECA: This model extends WheatNet by integrating both the TPSA and ECA (Efficient Channel Attention) modules in parallel, allowing for improved feature representation and classification performance.

**Figure 7:**
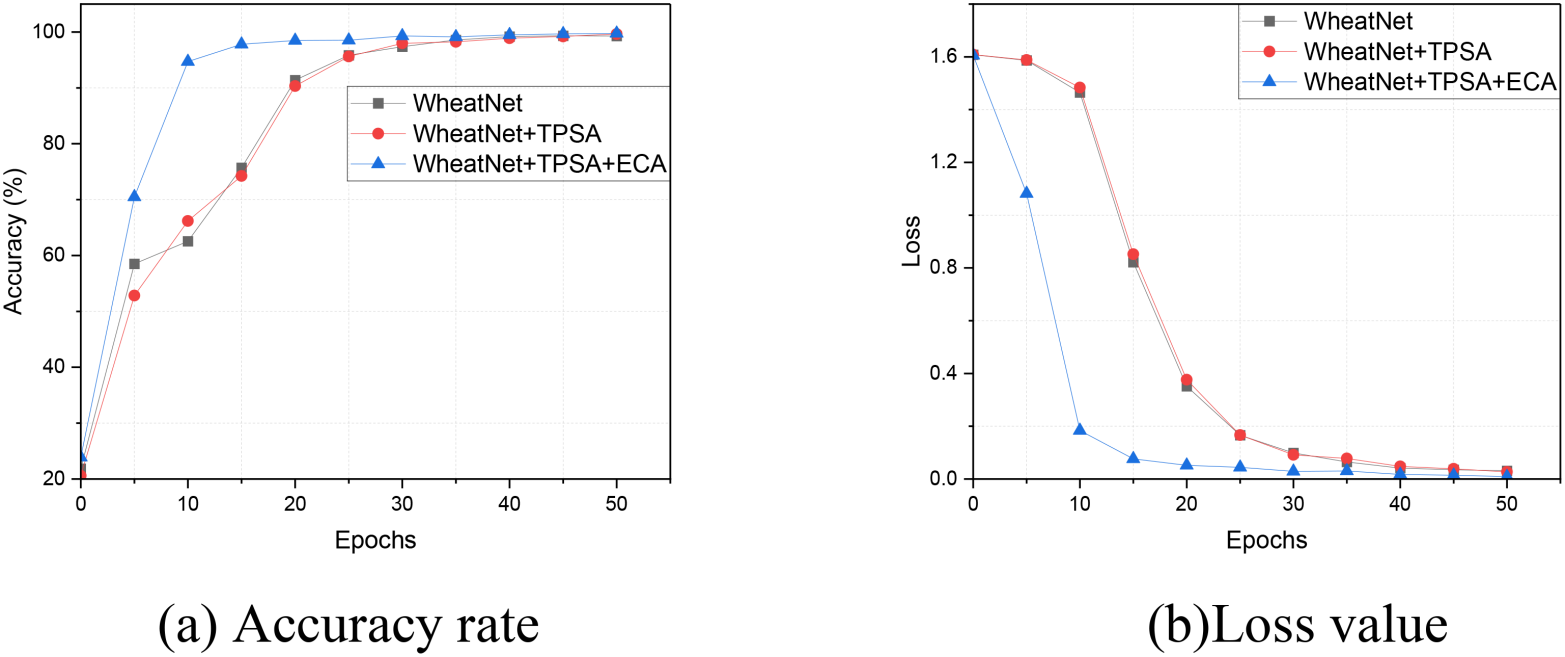
The Ablation Experiment Results of Accuracy and Loss on Training Dataset. **(A)** Accuracy rate, **(B)** Loss value.

These experiments provide compelling evidence for the effectiveness of the LWheatNet model. By systematically integrating enhanced modules under controlled conditions, we can observe the progressive improvements in performance metrics at each stage of development. Various metrics, including accuracy and loss, are employed to illustrate these performance enhancements. The results of the ablation experiments, which detail accuracy and loss on the training dataset, are presented in **Figure 7**. Additionally, the performance metrics on the test dataset are summarized in **Table 4**, further reinforcing the improvements achieved through the integration of the Two-Path Spatial Attention (TPSA) and Efficient Channel Attention (ECA) components.

**Table 4:** Results of ablation experiment on test dataset.

Model	Loss	Accuracy (%)	Precision_w_ (%)	Recall_w_ (%)	F1_w_ (%)	Params
WheatNet	0.0934	96.57	96.89	96.57	96.60	**1.26M**
WheatNet+TPSA	0.0641	97.38	97.53	97.38	97.38	1.33M
WheatNet+TPSA+ECA(**LWheatNet**)	**0.0562**	**98.59**	**98.61**	**98.59**	**98.59**	1.33M

Bold values indicate the best values for each evaluation metric.

From **Figure 7**, it is evident that LWheatNet exhibits the fastest convergence speed among the models evaluated within the 50 iterations. This rapid convergence is characterized by a significant reduction in loss and a corresponding improvement in accuracy across successive iterations. The swift decline in loss indicates that LWheatNet effectively learns from the training data, quickly adapting to the underlying patterns in the wheat classification task. Simultaneously, the increase in accuracy reflects the model’s ability to make correct predictions more consistently as training progresses. This efficient learning process not only highlights LWheatNet’s robustness but also underscores its potential for practical applications, where rapid model training can lead to quicker deployment in real-world scenarios.

**Table 4** provides a detailed comparison of the performance metrics for three different wheat classification models, elucidating their respective strengths and improvements. WheatNet serves as the baseline model, demonstrating solid performance with a loss of 0.0934 and an accuracy of 96.57%. This establishes a foundational benchmark for evaluating the subsequent models. The introduction of the Two-Path Spatial Attention (TPSA) component in the WheatNet+TPSA model results in a significant enhancement in performance. The loss decreases to 0.0641, while accuracy improves to 97.38%. This improvement indicates that TPSA effectively directs the model’s attention to relevant spatial features, leading to better classification outcomes. Further advancements are observed in the LWheatNet model, which integrates both the TPSA and Efficient Channel Attention (ECA) components. LWheatNet achieves the best overall performance among the models evaluated, with a loss of 0.0562 and an impressive accuracy of 98.59%. Additionally, the precision, recall, and F1 score for this model are all approximately 98.59%, reflecting a highly effective and balanced approach to classification tasks. Importantly, the parameter count for LWheatNet remains consistent with that of WheatNet+TPSA at 1.33 million parameters. This indicates that the integration of the ECA component does not significantly increase the model’s complexity, allowing for enhanced performance without a corresponding rise in computational demands.

Overall, the results demonstrate that the combination of TPSA and ECA attention mechanisms leads to substantial improvements in model efficacy while maintaining efficiency in terms of parameter count. The progression from WheatNet to WheatNet+TPSA and finally to LWheatNet (WheatNet+TPSA+ECA) illustrates clear enhancements in performance metrics, including lower loss and higher accuracy, precision, recall, and F1 scores. The addition of the TPSA and ECA components enhances the model’s effectiveness without a significant increase in parameters, making LWheatNet the most efficient and high-performing model in this comparison.

### 4.4 Comparative experiments

Comparative experiments are specifically designed to assess and compare the performance of distinct models. In this paper, we utilize the wheat seed dataset to compute the performance of LWheatNet. We train both widely recognized models and the LWheatNet across several measures to highlight the superior performance of the proposed model.

To further establish LWheatNet’s predominance, it is benchmarked against five other models: AlexNet (Krizhevsky et al., 2017), VGG16 (Simonyan and Zisserman, 2014), MobileNetV2 (Sandler et al., 2018), MobileNetV3 (Howard et al., 2019) and ShuffleNet V2 (Ma et al., 2018).

AlexNet and VGG16 are both seminal architectures in the field of deep learning, particularly in image classification tasks. AlexNet was a pioneer in demonstrating the power of deep learning and the use of GPUs for training, introducing key concepts like ReLU activations and dropout layers. However, its complexity and computational demands make it less practical for some applications. VGG16 built on the success of AlexNet by using smaller convolution filters and deeper networks to achieve higher accuracy. Its simplicity and high performance make it a go-to model for transfer learning, but it is also computationally intensive and has a large number of parameters, leading to potential inefficiencies.

MobileNetV2, MobileNetV3, and ShuffleNetV2 are all designed with efficiency in mind, making them suitable for mobile and embedded applications. MobileNetV2 intro-duces inverted residuals and linear bottlenecks to balance efficiency and performance, but may require more complex implementation.MobileNetV3 builds on MobileNetV2 by incorporating state-of-the-art techniques like squeeze-and-excitation modules and neural architecture search, offering improved efficiency and performance at the cost of increased complexity.ShuffleNetV2 focuses on optimizing the balance between computation and memory access cost using channel shuffle operations, offering a simpler and highly efficient architecture.

**Table 5** provides a detailed overview of the parameters used for the comparative models, while **Table 6** presents the experimental results on the test dataset.

**Table 5:** Parameters of comparative models.

Model	Parameters	Value
AlexNet	Batch_Size	16
	Epoch	50
	Learning_Rate	1e-04
	Dropout	0.3
	Hidden layer	3
	Conv layer	16
	Conv kernel	3×3
	Feature dimension	4096
VGG16	Batch_Size	16
	Epoch	50
	Learning_Rate	1e-03
	Dropout	0.3
	Hidden layer	3
	Conv layer	5
	Conv kernel	3×3, 5×5,11×11
	Feature dimension	4096
MobileNetV2	Batch_Size	16
	Epoch	50
	Learning_Rate	1e-03
	Depthwise separable layer	20
	Conv layer	30
	Conv kernel	3×3
	Feature dimension	1280
MobileNetV3	Batch_Size	16
	Epoch	50
	Learning_Rate	1e-03
	Depthwise separable layer	15
	Conv layer	20
	Conv kernel	3×3
	Feature dimension	1024
ShuffleNet V2	Batch_Size	16
	Epoch	50
	Learning_Rate	1e-03
	Dropout	0.4
	Conv kernel	1×1,3×3
	Feature dimension	1024

**Table 6:** Comparative model experimental results on wheat test set.

Model	Loss	Accuracy (%)	Precision_w_ (%)	Recall_w_ (%)	F1_w_ (%)	Params
AlexNet	0.5379	86.9	87.16	86.9	86.93	14.59M
VGG16	0.7823	81.25	81.31	81.25	81	134.28M
MobileNet V2	0.4125	86.9	87.17	86.9	86.76	2.23M
MobileNet V3	0.2933	88.31	88.76	88.31	88.24	1.52M
ShuffleNet V2	0.0934	96.57	96.89	96.57	96.60	**1.26M**
LWheatNet	**0.0562**	**98.59**	**98.61**	**98.59**	**98.59**	1.33M

Bold values indicate the best values for each evaluation metric.

**Table 6** provides a comprehensive comparison of various models employed for the wheat seed classification task. LWheatNet is identified as the most effective model, achieving an accuracy of 98.99% and a minimal loss of 0.0413, while maintaining a parameter count of 1.33 million. This performance highlights LWheatNet’s capability in balancing accuracy and computational efficiency. MobileNet V2 and MobileNet V3 also exhibit commendable results, surpassing the performance of AlexNet and VGG16. Notably, MobileNet V3 achieves an accuracy that is 1.41 percentage points higher than that of AlexNet and 7.06 percentage points higher than VGG16, demonstrating its efficacy in the classification task while utilizing fewer parameters and exhibiting lower loss values. ShuffleNet V2 further distinguishes itself through its efficiency, attaining an accuracy of 98.79% with only 1.26 million parameters. This makes it one of the most resource-efficient models in the comparison, although it records a slightly higher loss of 0.0545 compared to LWheatNet. Conversely, VGG16 and AlexNet, despite their larger parameter sizes, do not yield superior performance in this context. The findings suggest that the increased complexity of these models does not correlate with enhanced accuracy, indicating that model architecture and design are critical factors in determining performance.

In conclusion, LWheatNet emerges as the most robust model for wheat seed classification, offering an optimal balance of accuracy and loss. While ShuffleNet V2 is notable for its efficiency, LWheatNet’s superior precision and recall metrics render it the preferred choice for this application. Overall, the analysis underscores the importance of selecting models that effectively integrate accuracy and computational efficiency, with LWheatNet and ShuffleNet V2 being the most suitable candidates for high-performance classification tasks in this domain.

**Figure 8:**
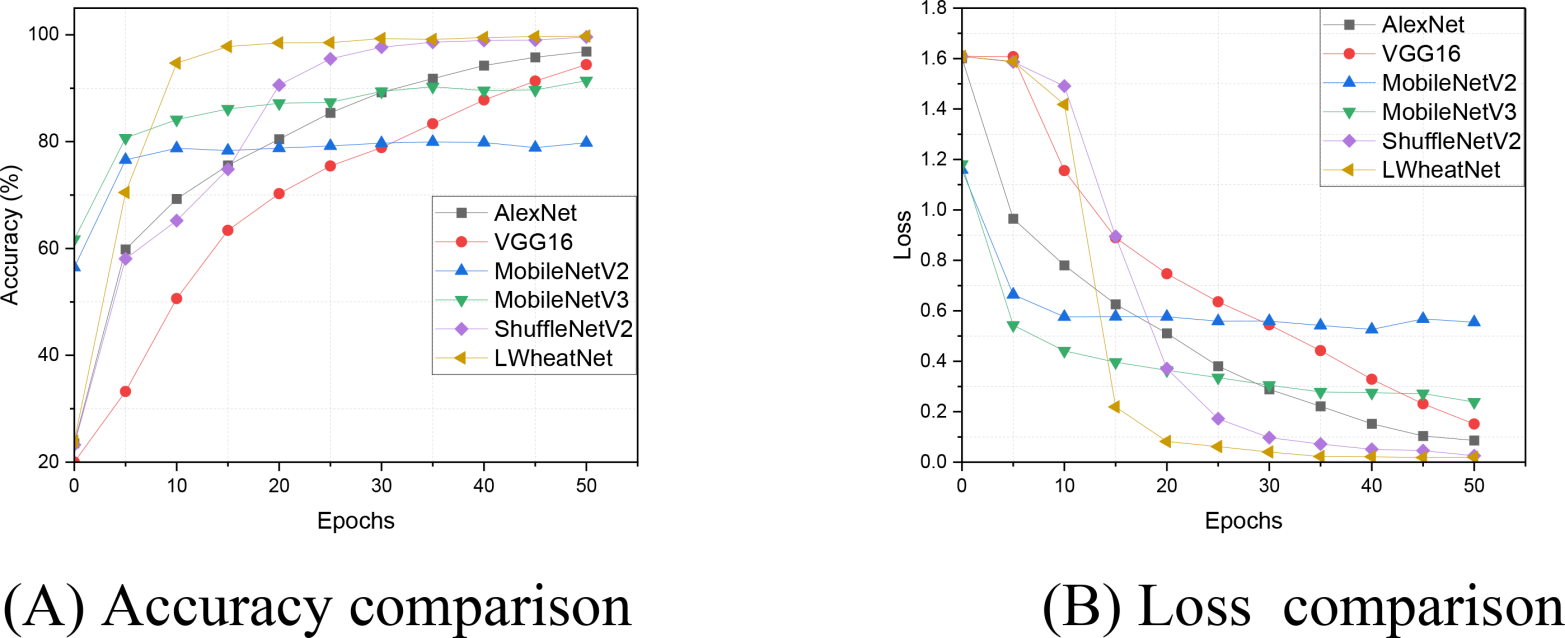
The accuracy and loss of comparative models on training set. **(A)** Accuracy comparison, **(B)** Loss comparison.

To evaluate the convergence efficiency of LWheatNet in relation to other models, we analyzed the changes in training loss and accuracy over several training rounds. **Figure 8** illustrates a detailed comparison of accuracy and loss across 50 epochs using the wheat seed training dataset. This comparison allows us to observe the convergence rates of each model towards optimal performance. The results highlight LWheatNet’s ability to achieve high accuracy and low loss efficiently within a limited number of epochs, suggesting its effectiveness in this context.

As shown in **Figure 8**, LWheatNet exhibits a notably faster convergence rate compared to other models, while maintaining a level of classification accuracy that is comparable. Additionally, LWheatNet achieves a higher classification accuracy at similar convergence rates, suggesting its enhanced convergence efficiency for the task of wheat seed image classification. These findings indicate that LWheatNet is capable of rapidly and accurately learning from the training data, positioning it as a potentially effective model for this specific application. The swift convergence may enhance the model’s practical applicability in real-world scenarios and suggests the possibility of more efficient training processes. Overall, these characteristics may contribute to LWheatNet’s potential role in improving precision agriculture through advanced image classification techniques.

### 4.5 Classification experiment

**Figure 9:**
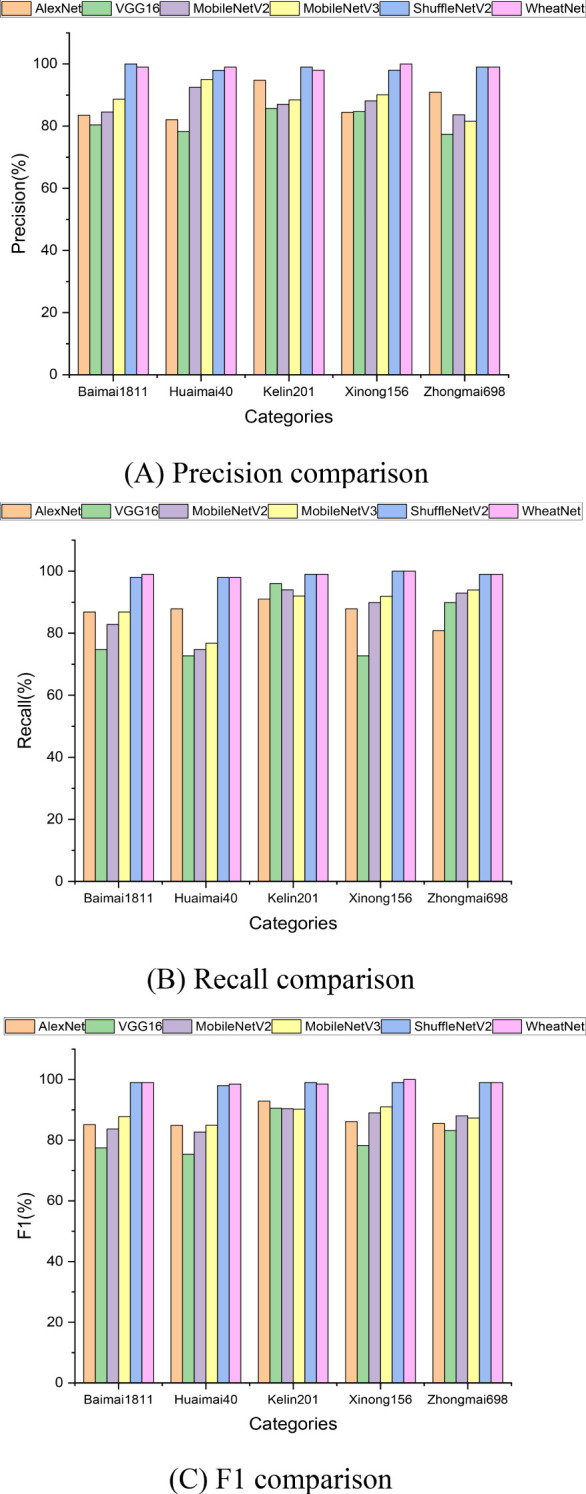
Comparative model experimental results of precision, recall and F1. **(A)** Precision comparison, **(B)** Recall comparison, **(C)** F1 comparison.

To explore the differences and performance impacts among the models, we performed a thorough analysis of Precision, Recall, and F1-score values for each model across various categories. The results from the wheat seed test dataset are illustrated in **Figure 9**. This analysis aims to highlight the relative strengths and weaknesses of each model, offering a clearer understanding of their effectiveness in classifying the wheat seed data.

**Figure 9** illustrates that the categories Kelin201 and Baimai1811 achieve relatively high scores in both Recall and F1 metrics, reflecting strong classification performance for these wheat seed images. In contrast, the categories Huaimai40 and Xinong156 display lower scores in Recall and F1, indicating potential challenges in feature extraction for these specific types of images. When assessing the precision metric, it is noteworthy that all models, except for VGG16, perform well in the 5-class classification task. This observation suggests that deeper neural networks with larger training parameters may not necessarily yield better classification performance for the wheat seed dataset. In terms of the F1 metric, LWheatNet and ShuffleNet V2 demonstrate the best classification performance. LWheatNet, in particular, benefits from the integration of a mixed attention mechanism, which enhances its ability to extract both local and global features from wheat seed images. This comprehensive feature extraction capability plays a significant role in LWheatNet’s superior performance.

**Figure 10:**
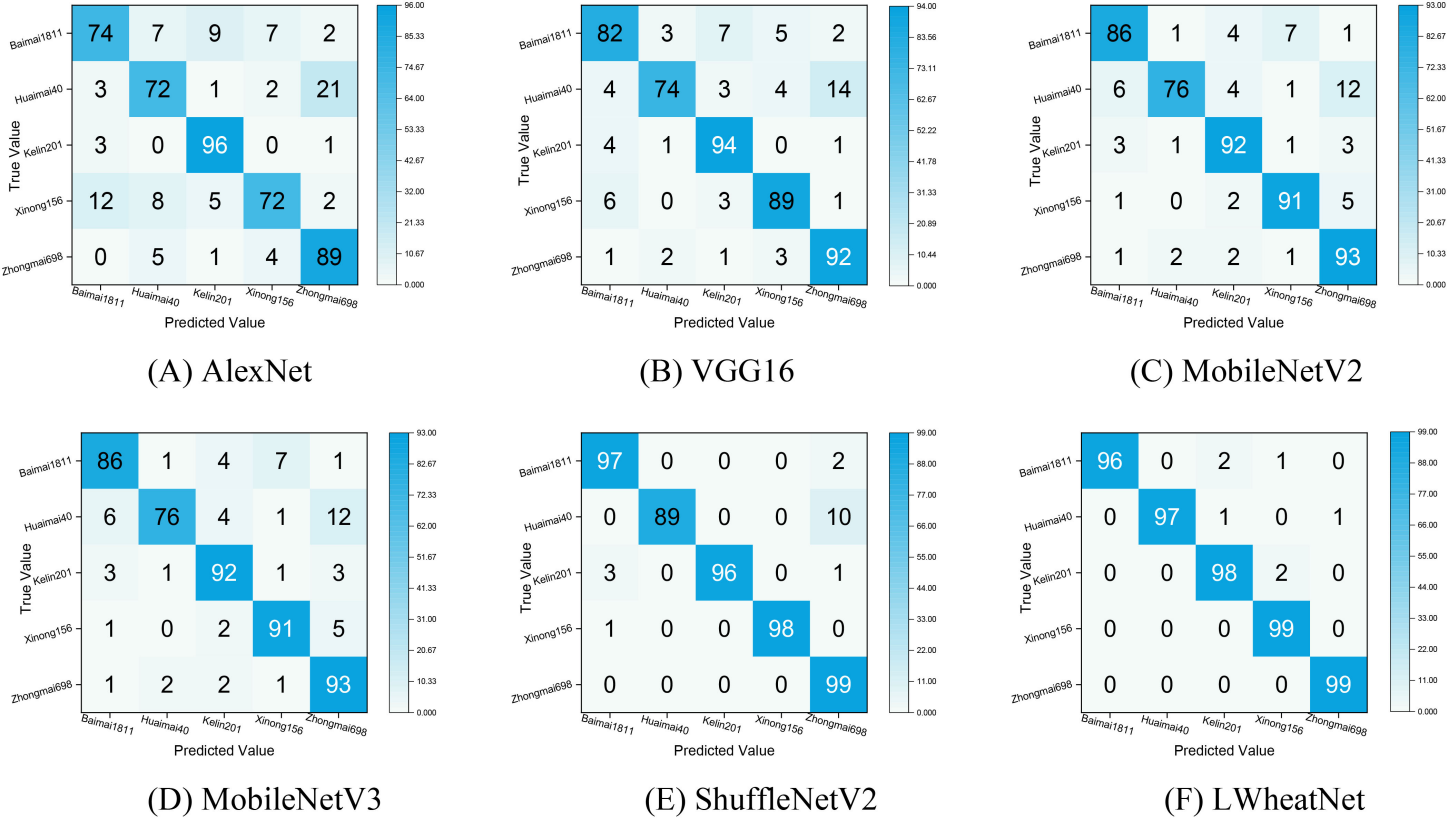
Confusion matrix of classification effect of each model. **(A)** AlexNet , **(B)** VGG16, **(C)** MobileNetV2, **(D)** MobileNetV3, **(E)**ShuffleNetV2, **(F)**LWheatNet.

To gain a more nuanced understanding of each model’s classification performance across different categories, we constructed a confusion matrix based on the classification results from the test set. The confusion matrix, depicted in **Figure 10**, provides a clear representation of the classification outcomes, with rows corresponding to the true categories and columns corresponding to the predicted categories. The diagonal elements indicate the number of correctly identified wheat seed images, while the off-diagonal elements reflect the number of misclassified images. This visual representation serves as an effective tool for assessing the strengths and weaknesses of the models in distinguishing between various categories of wheat seeds.

**Figure 10** illustrates that LWheatNet and ShuffleNet V2 achieve the lowest misclassification rates, demonstrating strong performance in classifying the five categories of wheat seeds. The confusion matrices for the AlexNet and VGG16 models indicate a higher frequency of misclassifying Xinong156 as Baimai1811. This misclassification may stem from the notable similarity in the external appearance of these two wheat grain types, which could have limited the models’ ability to extract sufficiently distinct features for accurate differentiation. In contrast, MobileNetV2 and MobileNetV3 show a significant improvement in reducing the misclassification rate for Xinong156, suggesting that these models are better equipped to distinguish between these visually similar categories.

Overall, the confusion matrix highlights that LWheatNet exhibits a higher level of accuracy in identifying various wheat seed classifications compared to the other models. This visualization underscores LWheatNet’s effectiveness in accurately categorizing a diverse range of wheat seed images, indicating its robustness and reliability for this specific application.

### 4.6 Generalization experiment

**Figure 11:**
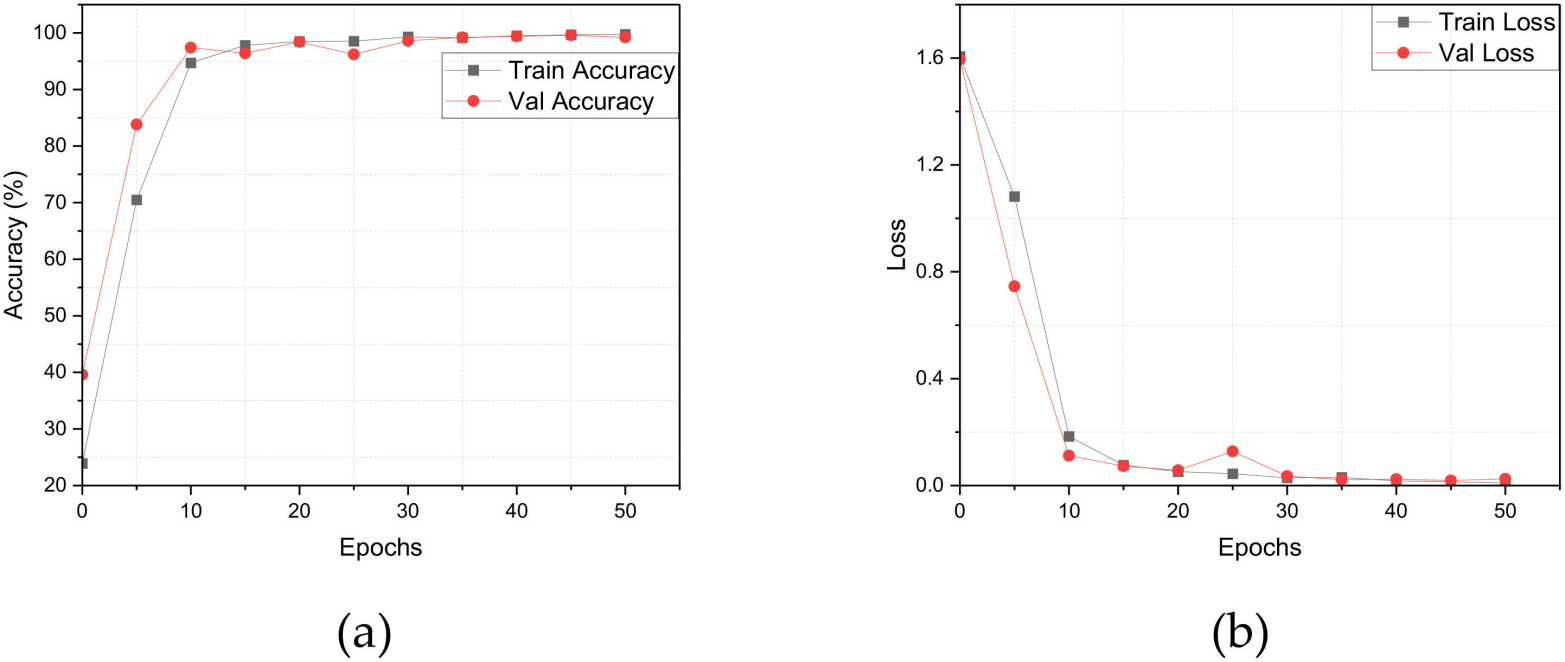
Comparison of accuracy rate and Loss value. **(A)** Accuracy, **(B)** Loss.

To evaluate the generalization capability of LWheatNet, we conducted a comparative analysis of accuracy and loss rates across the training and validation sets of the wheat seed dataset over a span of 50 epochs. The findings from this analysis are presented in **Figure 11**.

The analysis of **Figure 11** reveals that the training accuracy exhibits a steady upward trend over the course of 50 iterations, suggesting continuous improvements in the model’s performance on the training set. In contrast, the validation accuracy displays minor fluctuations, which may indicate variations in the model’s performance on unseen data at different stages of training. This variability suggests a dynamic nature in the model’s generalization ability as it adapts throughout the training process.

In terms of the Loss metric, the training set’s loss rate consistently decreases over the 50 iterations, reflecting the model’s effective adjustments to parameters aimed at minimizing training loss. This consistent reduction points to an enhancement in the model’s fitting capability. Conversely, the validation set’s loss rate shows slight fluctuations, which may arise from the model’s varying fit to the validation data at different stages of training. This behavior underscores the ongoing challenge of maintaining a balance between preventing overfitting and improving generalization ability as training progresses.

## 5 Discussion

The paper introduces a lightweight classification model, LWheatNet. This model combines the mixed attention mechanism with stacked inverted residual convolutional networks. To validate the proposed model, we constructed an image dataset encompassing five categories of wheat seeds. The experimental results demonstrate that LWheatNet enhances feature extraction, improves classification accuracy, and operates with minimal parameters.

The LWheatNet model successfully addresses some of the key challenges in wheat seed variety classification by providing a high-performance, lightweight solution. The construction of a dedicated dataset and the development of an efficient model architecture represent significant advancements in this field. LWheatNet’s ability to deliver high ac-curacy while maintaining a small model size makes it particularly valuable for real-time applications in agriculture, especially in resource-limited settings.

While the LWheatNet demonstrates significant advancements in wheat seed image classification, it is not without its limitations. One shortcoming of the LWheatNet model is that the dataset used for classification contains only five wheat varieties. To ensure the robustness and generalizability of the model, it is essential to validate its performance on a dataset with a larger number of categories in future studies. Another limitation arises from the way the dataset is divided. The training, validation, and test sets are randomly split, and since the wheat seeds are collected from two different angles, the same seeds appear in the dataset from different perspectives. This overlap may introduce fluctuations in the analysis results and affect the overall performance evaluation of the model.

## 6 Conclusions

This study aims to address some of the challenges associated with classifying wheat seed images, specifically long processing times, high computational demands, and low classification accuracy. To this end, we introduce a lightweight classification model called LWheatNet. The key contributions of this research are outlined as follows:

1. In response to the limited availability of publicly accessible datasets, we created a comprehensive dataset comprising single-seed images from five different wheat varieties. This robust database serves as an essential resource for the development and validation of deep learning models in the realm of wheat seed classification, benefiting both researchers and practitioners.
2. We proposed LWheatNet, a lightweight convolutional neural network that integrates a mixed attention module with stacked inverted residual convolutional networks. This model not only enhances the classification performance of wheat images but also maintains a relatively small number of parameters, making it suitable for deployment in resource-constrained environments, such as mobile devices or edge computing.
3. We conducted a series of comparative, classification, and generalization experiments to demonstrate the efficacy of our proposed model. Using various evaluation metrics, we achieved an accuracy of 98.59% and a loss of 0.0562. The results indicate that LWheatNet outperforms several traditional network models, achieving high classification accuracy while requiring minimal computational resources.

The findings of this study hold significant implications for research and practical applications in agriculture and food science. By providing an efficient and accurate tool for wheat seed classification, LWheatNet could facilitate improved crop management, enhance breeding programs, and contribute to overall food security. The ability to classify wheat seeds quickly and accurately may support better decision-making processes in agricultural practices, enabling farmers and agronomists to optimize their operations.

Looking forward, future research could focus on further optimizing the LWheatNet architecture to reduce computational complexity and model size while maintaining or even enhancing classification accuracy. Techniques such as model pruning, quantization, and knowledge distillation could be explored to create a more efficient and compact model without sacrificing performance. Additionally, expanding the dataset to include a broader variety of wheat species and potentially other crops would improve the model’s generalization capabilities, making it a more versatile tool for agricultural research and practice. This approach could ultimately contribute to advancements in precision agriculture, where tailored solutions can be developed based on accurate seed classification.

## Data Availability

The datasets presented in this study can be found in online repositories. The names of the repository/repositories and accession number(s) can be found below: https://github.com/guoxiaojuanhist/Wheat-seed-dataset.
